# Marine sponge‐derived smenospongine preferentially eliminates breast cancer stem‐like cells via p38/AMPKα pathways

**DOI:** 10.1002/cam4.1640

**Published:** 2018-07-07

**Authors:** Jie Tang, Wei Wu, Fan Yang, Liyun Liu, Zhen Yang, Li Liu, Weizhuo Tang, Fan Sun, Houwen Lin

**Affiliations:** ^1^ Research Center for Marine Drugs State Key Laboratory of Oncogenes and Related Genes Department of Pharmacy Ren Ji Hospital School of Medicine Shanghai Jiao Tong University Shanghai China

**Keywords:** AMPKα, breast cancer, cancer stem‐like cells, p38, smenospongine

## Abstract

Breast cancer stem cells (CSCs) have been postulated as responsible for therapeutic failure of breast cancer. Novel agents effectively targeting breast CSCs are urging to be discovered to overcome cancer relapse and metastasis. We recently established a CSC‐like model through ectopic expression Nanog, a core pluripotency factor, in breast cancer cells and validated induced CSC‐like (MCF7‐Nanog) model acquired stem‐like properties. Using this model, we found that smenospongine (Sme), a natural sesquiterpene aminoquinone isolated from marine sponge *Spongia pertusa* Esper, preferentially inhibited the induced CSC‐like cells proliferation by inducing G0/G1 arrest and intrinsic apoptosis via increasing the phosphorylation level of p38 and AMPKα. Importantly, Sme exhibited the ability to abrogate CSC‐like cells associated with a downregulation of stem cell markers including Nanog, Sox2, and Bmi1. Functionally, Sme inhibited the ability of MCF7‐Nanog cells to form tumor sphere in vitro and develop tumor in vivo. Significant antitumor effects are observed in Sme‐treated mouse xenograft tumor models, with no apparent toxicity to mice. Taken together, our findings provide a CSC‐like model to identify novel CSC‐targeting drugs and identify Sme as a candidate natural agent for treatment of breast cancer.

## 1 INTRODUCTION

Breast cancer is the most common malignancy diagnosed in women and the leading cause of cancer‐related death among women in many countries.^1^, ^2^ Emerging evidence in recent years has demonstrated the existence of breast cancer stem cells (CSCs) that plays a critical role in oncogenesis, progression, relapse, and metastasis.^3^, ^4^, ^5^ CSCs are usually resistant to conventional cancer treatments such as chemotherapeutic agents and radiation ultimately resulting in treatment failure.^6^, ^7^ Therefore, the exploring of novel agents that eliminate CSCs might effectively eradicate breast cancer and increase survival rate.

As many screening technologies could facilitate the identification of agents targeting CSCs, however, screening for agents with CSCs‐specific toxicity requires bulk populations of stable and homogeneous CSCs in vitro. Breast CSCs enrichment is rapidly lost during in vitro culture because of incompatible with the tumor microenvironment in vivo.^7^ Recently, several new human CSCs models were established to identify compounds that can selectively target CSCs.^8^, ^9^, ^10^ Nanog is a core transcription factor firstly discovered in embryonic stem cells (ESCs) with essential functions in maintenance of self‐renewal and pluripotency of ESCs.^11^, ^12^ In recent years, the relationship between Nanog and cancer has received increased attention. Nanog was demonstrated to enhance the tumorigenicity both in vivo and in vitro and contribute to tumorigenesis, metastasis, recurrence, and therapeutic resistance.^13^ In oral squamous cell carcinoma and colorectal cancer patients, overexpression of Nanog was strongly correlated with poor prognosis, indicating Nanog may be a marker for survival prognosis.^14^, ^15^ Interestingly, overexpression of Nanog has been shown to result in an enrichment of CSC‐like population within established ovarian cancer cell lines.^16^ We demonstrate here that the involvement of Nanog in breast cancer cells exhibits much higher sphere‐forming ability, an increased drug resistance, and more tumorigenicity in vivo, indicating acquired CSC‐like properties. These cells, termed induced cancer stem cell‐like cells, were used to screen agents with selective toxicity for breast CSCs.

Furthermore, we reported that smenospongine (Sme), a sesquiterpene aminoquinone isolated from the marine sponge *Spongia pertusa* Esper,^17^ preferentially inhibited the growth of breast cancer stem‐like cells in vitro and in vivo without overtly toxicity on body weight of mice. Our findings suggested that Sme induced G0/G1 arrest and intrinsic apoptosis. The inhibitory effect of Sme was antagonized by decreasing the phosphorylation level of p38 and AMPKα. Collectively, our study highlights Sme as a potential agent for breast cancer therapies and, additionally, provides a useful method for future exploration of novel anti‐CSCs drugs.

## 2 MATERIALS AND METHODS

### 2.1 Isolation and Identification of Sme

Sme was isolated from the marine sponge *Spongia pertusa* Esper (collected in the South China Sea) and its structure was determined previously in our laboratory.^17^ As shown in Fig. S1, HPLC analysis revealed that the purity of Sme is over 98%.

### 2.2 Cell line and cell culture

Human breast cancer cell line MCF7, human mammary epithelial cell line HBL100 and human bronchial epithelial cell line 16HBE were purchased from the Shanghai cell bank, Chinese Academy of Sciences (Shanghai, China). All cells were grown in DMEM medium (Gibco, Grand Island, NY, USA) supplemented with 10% FBS (Gibco), 100 units/mL penicillin, 100 μg/mL streptomycin, and 200 U/mL recombinant insulin (Novo Nordisk, Copenhagen, Denmark).

### 2.3 Lentivirus generation and infection

Plasmid vectors encoding Nanog cDNA was purchased from GenePharma (Shanghai, China). Lentivirus production was described briefly as follows. 293T cells were seeded onto a 15 cm culture dish. After cultured overnight, cells were cotransfected with pGag/Pol, pRev, pVSV‐G, and lentiviral vector pGMLV‐PA6 containing Nanog genes for 6 hours, supplemented with 300 μL RNAi‐Mate. Then the medium was replaced with DMEM medium and cells were cultured for another 72 hours. The virus‐containing medium was collected and enriched, then used for ectopic expression of Nanog in MCF7 cells. MCF7 (3 × 10^4^) cells were seeded in a 12‐well plate 1 day before transfection. Lentivirus were diluted to the desired multiplicity of infection and then added to cells in the presence of 1 mL polybrene per well. After infected lentiviral for 24 hours, virus solution was replaced with complete medium and cells were cultured for another 2 days. Puromycin (Sigma‐Aldrich, St Louis, MO, USA) selection was performed to kill the mock‐transfected cells and stable clones were selected and cultured for further analysis.

### 2.4 Quantitative real‐time PCR

Total RNA was extracted using RNA simple total RNA kit (TIANGEN Biotech Co. Ltd., Beijing, China) and used to synthesize cDNA using PrimeScript^™^ RT reagent Kit (Perfect Real Time) (Takara Bio, Kusatsu, Japan) according to the manufacturer's instructions. Relative mRNA was determined by quantitative real‐time PCR using SYBR^®^ Premix Ex Taq^™^ II (Tli RNaseH Plus) (Takara Bio) with β‐actin mRNA level as a control. The primers for amplification were synthesized by Sangon Biotech (Shanghai, China) and present in Table S1.

### 2.5 Western blotting analysis

Cells were lysed on the ice with RIPA buffer containing protease and phosphatase inhibitor cocktails (MCE Co., Ltd, Shanghai, China) and protein concentrations were determined by a BCA protein assay kit (Beyotime Biotechnology, Suzhou, China). Equal loading of proteins were separated by 6‐15% SDS‐PAGE and transferred to a PVDF membrane (Millipore, Billerica, MA, USA), followed by blocked with 5% nonfat milk. Subsequently, membranes were orderly incubated with primary antibodies (Cell Signaling Technology, Beverly, MA, USA) at 4°C overnight and HRP‐conjugated secondary antibody (Abcam, Cambridge, UK) for 1 hours. Protein bands were scanned with ECL western blotting detection system (General Electric Company, Andover, MA, USA).

### 2.6 Immunofluorescence staining

MCF7‐Nanog cells were plated in multiple glass‐bottom tissue culture plates. After cultured overnight, cells were fixed by 4% paraformaldehyde for 10 minutes. Then cells were washed three times with cold PBS and permeated with 0.4% Triton X‐100 for 15 minutes. Cells were blocked with 1% BSA and incubated with primary antibodies against Nanog, Sox2, or CD44 overnight. The secondary antibodies Alexa Fluor^®^488 goat anti‐mouse and Alexa Fluor^®^647 goat anti‐rabbit IgG (Invitrogen, Carlsbad, CA, USA) were incubated with cells for 1 hours. Nuclei were stained with DAPI. Laser confocal imaging (Leica SP8, Wetzlar, German) were used to visualize localization of Nanog, Sox2, and CD44 in cells.

### 2.7 Drug resistance assay

Cells were seeded in 96‐well plates. After cultured overnight, cells were treated with different concentrations of cisplatin, tamoxifen, gemcitabine, or paclitaxel (Sigma‐Aldrich) and incubated for 48 hours. CCK‐8 solution (Dojindo, Tokyo, Japan) were added to each well, and the absorbance was measured at 450 nm in a microplate reader (Molecular Devices, California, USA).

### 2.8 Sphere formation assay

Cells were plated in 24‐well ultra‐low‐attachment plates (Corning, Bedford, MA, USA) at a density of 5000 cells/mL and grown in DMEM‐F12 medium (Thermo Fisher Scientific, Rockford, IL, USA), supplemented with 2% B27 supplement (Invitrogen), 4 g/L BSA, 20 ng/mL of EGF, 20 ng/mL of bFGF (Invitrogen), and 4 mg/mL of heparin (Sigma‐Aldrich). When cells were cultured for 14 days, the numbers of spheres were photographed and counted.

### 2.9 Xenograft tumorigenicity assay

All of the animal studies and procedures were approved by Animal Care and Use Committee of Ren Ji Hospital, Shanghai, China (permission number: SYXK (Hu) 2016‐0009). Four‐ to five‐week‐old female NOD/SCID mice were purchased from Shanghai Slac Laboratory Animal Co., Ltd (Shanghai, China). Prior to cell injection, mice were injected estrogen (Sigma‐Aldrich) intraperitoneally every two days for a week. MCF7 cells and virus‐infected MCF7 cells were harvested, respectively, and cells (6 × 10^6^) were resuspended in PBS. MCF7 cells, MCF7‐Ctrl cells, and MCF7‐Nanog cells were admixed with matrigel (BD Biosciences, Mountain View, CA, USA) as 1:1, respectively, and then injected orthotopically in the mammary fat pad of NOD/SCID mice (five mice per group). The mice were monitored for tumor growth since injection and then killed with cervical dislocation on day 35 after injection. Subcutaneous tumors were removed from the mice, weighed, measured, and photographed.

### 2.10 Cell cycle assay

Cells were harvested after exposure to various concentrations of Sme for 48 hours and fixed with 70% ethanol at 4°C overnight. The cells were then stained with PI‐RNAse solution (BD Biosciences) for 30 minutes in the dark. Then the samples were analyzed cell cycle distribution by flow cytometer (BD Biosciences).

### 2.11 Cell apoptosis analysis

The apoptosis induced by Sme was detected by Annexin V‐FITC/Sytox Red double‐staining. Cells were treated with different concentrations of Sme and harvested followed by staining with Annexin V‐FITC (Thermo Fisher Scientific) in dark for 15 minutes and Sytox Red (Thermo Fisher Scientific). The samples were quantified using flow cytometer.

### 2.12 In vivo antitumor activity

MCF7‐Nanog cells (1 × 10^7^) mixed with matrigel were orthotopically injected in the mammary fat pad of four‐ to five‐week‐old female NOD/SCID mice in which estrogen was intraperitoneally injected every other day for three times prior to cell injection. Two weeks after injection, the mice were divided into two groups (six mice per group): control and Sme (20 mg/kg), followed by intraperitoneally injection with Sme every other day for 2 weeks. The body weight was monitored every day. At the termination of experiment, the mice were sacrificed and the tumors were excised, and fixed in 4% paraformaldehyde for the TUNEL assays and H&E staining.

### 2.13 TUNEL assays

In brief, paraformaldehyde‐fixed tumors were embedded in paraffin and sectioned at a thickness of 4 μm. Next, the slides were deparaffinized with xylene and ethanol, and incubated with proteinase K, followed by PBS wash for three times. TUNEL reaction mixture (Roche Diagnostics, Mannheim, Germany) were prepared and added to the slides, and slides were incubated for 1 hours in dark moist environment. Finally, after washed with PBS, the apoptotic cells on the tumors were visualized using the fluorescence microscopy (Nikon, Tokyo, Japan).

### 2.14 H&E staining

Following a hydration process, 4 μm paraffin‐embedded sections were orderly incubated with hematoxylin and eosin‐phloxine solution, dehydrated, and immersed in xylene. After mounted with neutral resin, the morphology of sections was visualized by the fluorescence microscopy (Nikon).

### 2.15 Statistical analysis

All experiments repeated at least three times and expressed as mean ± SD. The significances of different groups’ results were analyzed by Student's *t* test and ANOVA and *P* < .05 was considered as statistically significant.

## 3 RESULTS

### 3.1 Nanog overexpression enhances cancer stem‐like properties in MCF7 cells

**Figure 1:**
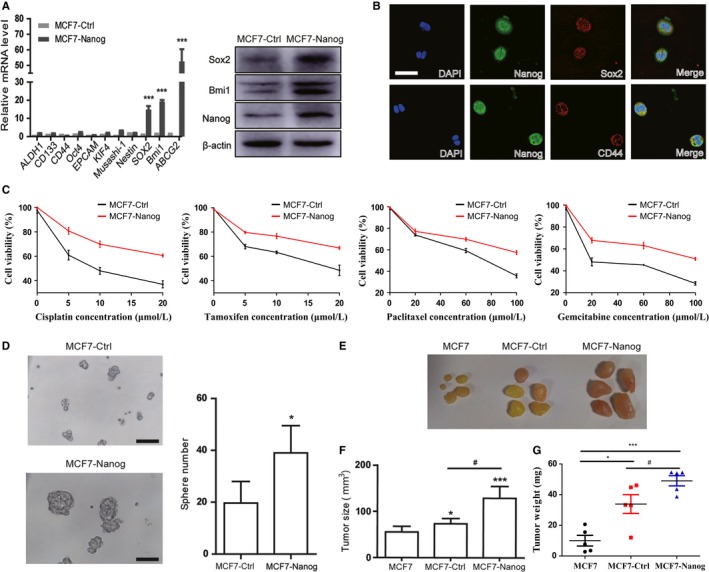
Nanog overexpression enhances cancer stem‐like properties in MCF7 cells. A, MCF7 cells were infected with lentiviral vectors encoding cDNA of Nanog (MCF7‐Nanog) or a control empty vector (MCF7‐Ctrl). Quantitative real‐time PCR and Western blotting were conducted to evaluate the expression of stem cell markers. Data are presented as mean ± SD. *** *P* < .001. B, Immunofluorescence staining was utilized for evaluating the colocation of Nanog, Sox2, and CD44 in MCF7‐Nanog cells. Scale bar, 400 μm. C, MCF7‐Nanog cells and MCF7‐Ctrl cells were treated with cisplatin, tamoxifen, paclitaxel, and gemcitabine for 72 hours. The cell viability was determined by CCK‐8 assay. D, MCF7‐Nanog cells and MCF7‐Ctrl cells were subjected to sphere formation assay. Scale bar, 100 μm. * *P *< .05. E, MCF7‐Nanog cells, MCF7‐Ctrl cells, and MCF7 cells (6 × 10^6^) were, respectively, injected into the mammary fat pad of four‐ to five‐week‐old female NOD/SCID mice orthotopically. 35 days after injection, tumors were surgically excised and MCF7‐Nanog cells generated larger tumors than MCF7‐Ctrl cells and MCF7 cells. F, G, The average volume and weight of the MCF7‐Nanog tumors were markedly increased than those of MCF7‐Ctrl and MCF7 tumors. * *P *< .05, *** *P *< .001 vs MCF7 group, # *P *< .05 compared with MCF7‐Ctrl group

Using lentiviral infection system, we produced stable cell lines (MCF7‐Nanog) from human breast cancer cells MCF7 with plasmid vectors encoding Nanog cDNA. An empty vector‐transfected control (MCF7‐Ctrl) was generated simultaneously. Quantitative real‐time PCR analysis showed that the mRNA level of stem cell‐specific markers, including Bmi1 and Sox2, were elevated in MCF7‐Nanog cells (Figure 1A). Western blotting analysis displayed an increased expression of Nanog, Bmi1, and Sox2 in MCF7‐Nanog cells (Figure 1A). Colocalization of Nanog and CD44, Nanog, and Sox2 in MCF7‐Nanog cells was confirmed by laser confocal imaging (Figure 1B).

Drug resistance and sphere formation are important characteristics of cancer stem cells. Our data showed that MCF7‐Nanog cells were more sustainable to conventional chemotherapeutic drugs including cisplatin, tamoxifen, paclitaxel, and gemcitabine (Figure 1C). Quantitative real‐time PCR also showed an enhanced expression of the ABC family of multidrug‐resistant genes ABCG2 (Figure 1A). Meanwhile as shown in Figure 1D, compared with the control group, MCF7‐Nanog cells possessed a greater ability to form sphere in serum‐free media, suggested that Nanog overexpression promoted the self‐renewal characteristic.

Given that cancer stem cells have a strong tumorigenic ability, we subsequently determined whether Nanog overexpression enhances tumorigenicity of MCF7 cells in vivo. MCF7‐Nanog, MCF7‐Ctrl, and MCF7 cells were, respectively, injected orthotopically in the mammary fat pad of four‐ to five‐week‐old female NOD/SCID mice in which estrogen was intraperitoneally injected every other day for three times prior to cell injection. Tumor growth was monitored every day and tumors in MCF7‐Nanog cells group were initiated earlier than those in MCF7‐Ctrl and MCF7 cells groups (Table S2). The tumors generated by MCF7‐Nanog cells were comparable to those of MCF7‐Ctrl cells and MCF7 cells 35 days after injection (Figure 1E). A marked increase in tumor volume and tumor weight (Figure 1F,G) was showed by tumors of the MCF7‐Nanog group, suggested that expression of Nanog promoted tumorigenic ability of MCF7 cells in vivo.

### 3.2 Sme preferentially inhibits MCF7‐Nanog and induces G0/G1 arrest

**Figure 2:**
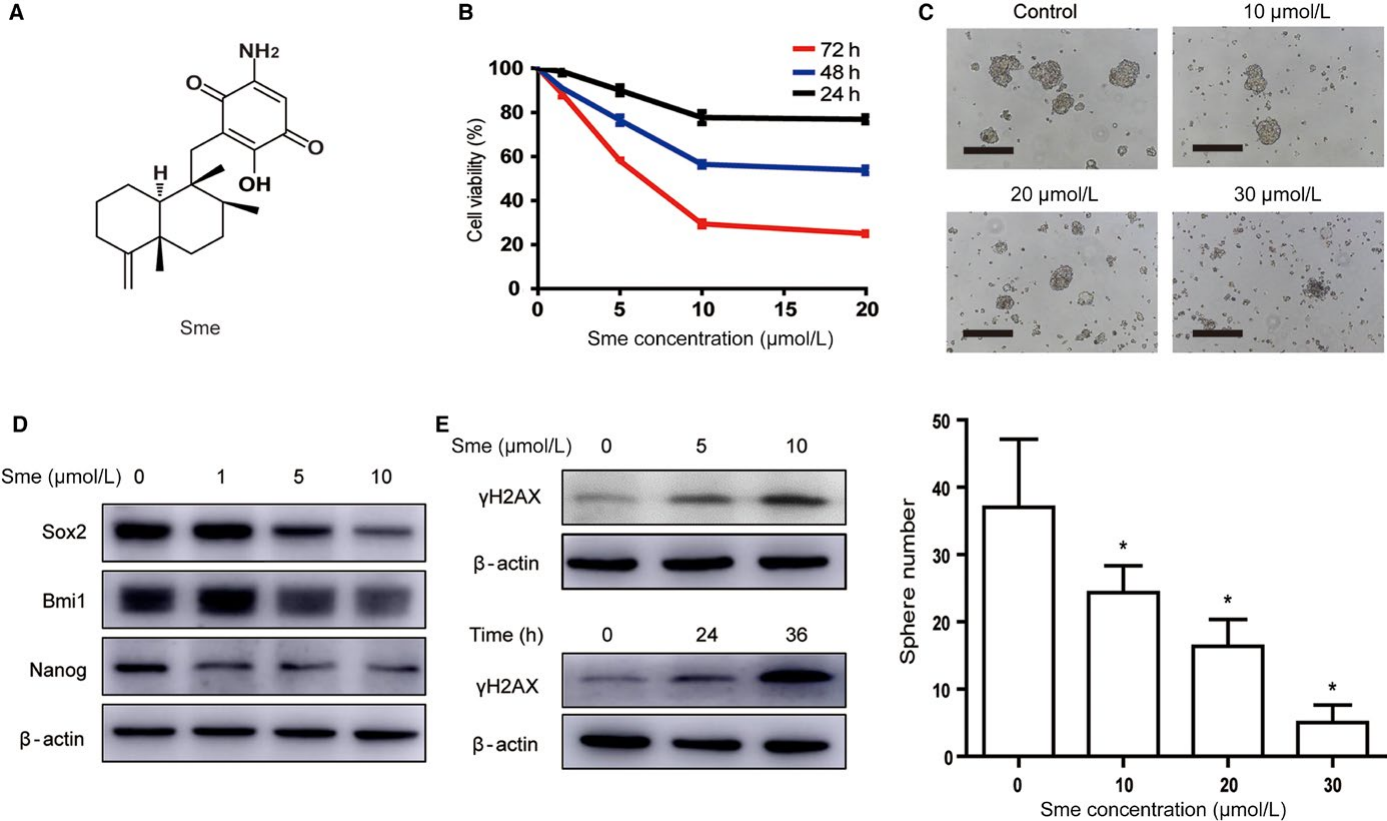
Sme preferentially inhibits MCF7‐Nanog cells proliferation and caused DNA damage. A, The chemical structure of Sme. B, Cytotoxicity of Sme to MCF7‐Nanog cells. Cells were incubated with Sme with different concentrations for indicated time points, and the viability was determined by CCK‐8 assay. C, MCF7‐Nanog cells were exposed to Sme for 72 hours and subjected to sphere‐forming assay. Scale bar, 100 μm. Data are presented as mean ± SD. * *P *< .05. D, Sme suppressed the expression of stemness‐related markers in MCF7‐Nanog cells. Western blotting was used to detect the expression of Nanog, Sox2, and Bmi1 after treatment with Sme for 36 h. E, The expression of γH2AX was decreased in dose‐ and time‐dependent manner after exposure to Sme

We investigated the growth inhibitory effect of conventional antitumor drugs, including tamoxifen, 4‐hydroxytamoxifen, cisplatin and paclitaxel, against MCF7‐Ctrl cells and MCF7‐Nanog cells. MCF7‐Nanog cells displayed resistance to all four antitumor drugs, with higher IC_50_ values than that of MCF7‐Ctrl cells (Table 1). However, Sme (Figure 2A) exhibited stronger suppressive effect on the proliferation of MCF7‐Nanog cells, indicating a preferential antitumor activity against cancer stem‐like cells (Table 1). Further, CCK‐8 assay demonstrated that Sme inhibited the viability of MCF7‐Nanog cells in a time‐ and concentration‐dependent manner (Figure 2B). Normal human mammary epithelial cell line HBL100 and human bronchial epithelial cell line 16HBE were selected to test for antiproliferative activity of Sme. Compared with the malignant cells, Sme exhibited a weaker growth inhibitory effect against the nonmalignant cells, with IC_50_ values of 21.30 ± 1.11 μmol/L and 30.88 ± 0.45 μmol/L (Figs S2 and S3). As shown in Figure 2C, Sme suppressed sphere‐forming ability of MCF7‐Nanog cells. Concomitantly, MCF7‐Nanog cells incubated with Sme exhibited a decrease in the expression of stem cell‐specific proteins, including Nanog, Bmi1, and Sox2 (Figure 2D). In conclusion, these data indicated that Sme inhibited proliferation of MCF7‐Nanog cells and might more effectively eliminate CSC‐like cells.

**Table 1:** IC_50_ values of MCF7‐Ctrl and MCF7‐Nanog cells

Chemicals	IC_50_ values (μmol/L)	
	MCF7‐Ctrl	MCF7‐Nanog
Tamoxifen	14.68 ± 1.47	24.43 ± 0.88
4‐Hydroxytamoxifen	5.84 ± 0.93	16.86 ± 1.46
Cisplatin	20.28 ± 1.45	28.81 ± 4.13
Pacitaxel	37.72 ± 2.23	55.12 ± 9.93
Sme	17.14 ± 1.84	6.06 ± 0.33

The failure of DNA damage repair might trigger cell cycle arrest to regulate cell cycle progress. Abnormal cell cycle distribution influences the proliferation, growth, and survival of cancer cells.^18^ To investigate whether Sme triggered DSBs, we detected the phosphorylated level of H2AX (γH2AX). As shown in Figure 2E, Sme dose‐ and time‐dependent enhanced the expression of γH2AX in MCF7‐Nanog cells, indicating that DNA damage involved in Sme‐induced antiproliferative activity.

**Figure 3:**
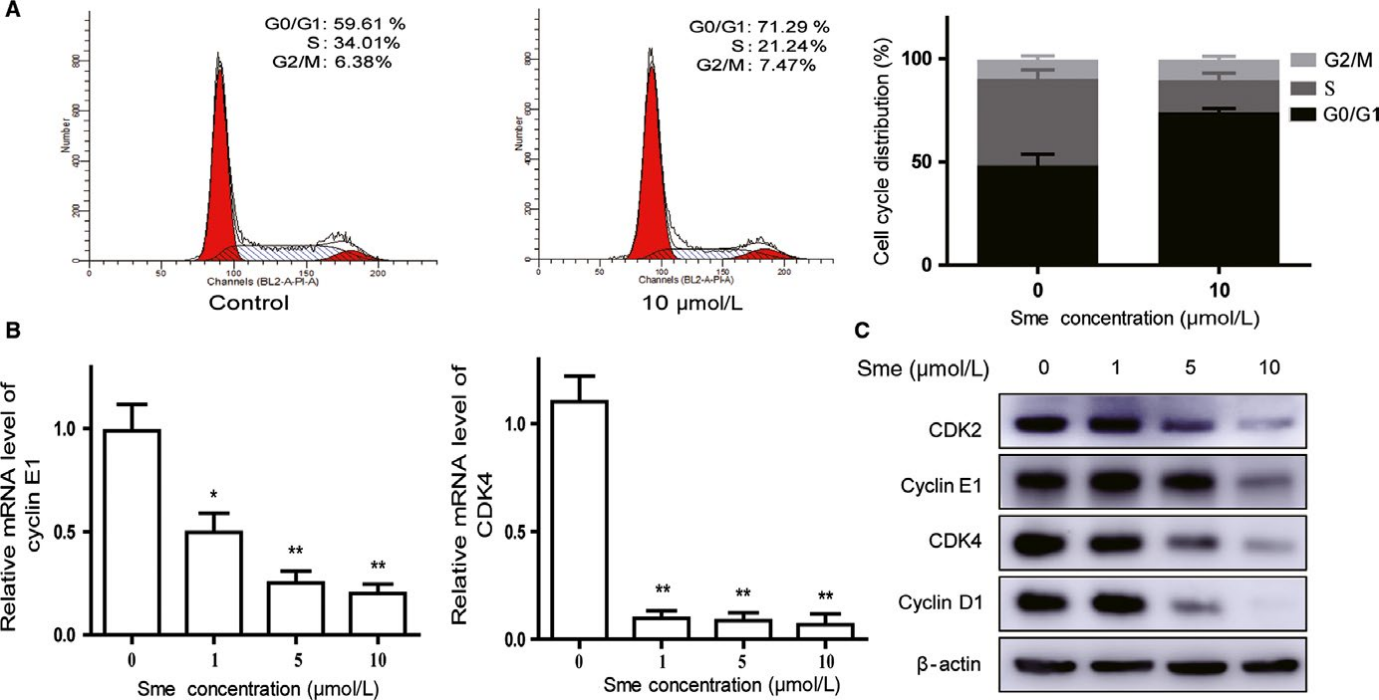
Sme induces G0/G1 arrest in MCF7‐Nanog cells. A, MCF7‐Nanog cells were treated with Sme (0 and 10 μmol/L) for 48 hours followed by flow cytometric assay. The histograms described the percentage of cell cycle distribution. B, C, The mRNA expression of CDK 4 and cyclin E1 was detected after exposed to Sme for 36 hours. The expression of G0/G1 phase‐related proteins was analyzed by Western blotting. Data are presented as mean ± SD. * *P *< .05, ** *P *< .01

Flow cytometry was used to evaluate cell cycle distribution. As shown in Figure 3A, MCF7‐Nanog cells were arrested at G0/G1 phase after treatment with 10 μmol/L Sme for 48 hours. Further quantitative real‐time PCR analysis showed that Sme significantly reduced the mRNA expression of cyclin E1 and cyclin‐dependent kinase 4 (CDK4) (Figure 3B). Meanwhile, western blotting analysis demonstrated that the expression of cyclin D1, cyclin E1, CDK2, and CDK4 were decreased in a dose‐dependent manner (Figure 3C). Taken together, these results indicated that Sme might induce G0/G1 arrest through modulating the expression of cell cycle regulatory molecules.

### 3.3 Sme induces apoptosis in MCF7‐Nanog cells

**Figure 4:**
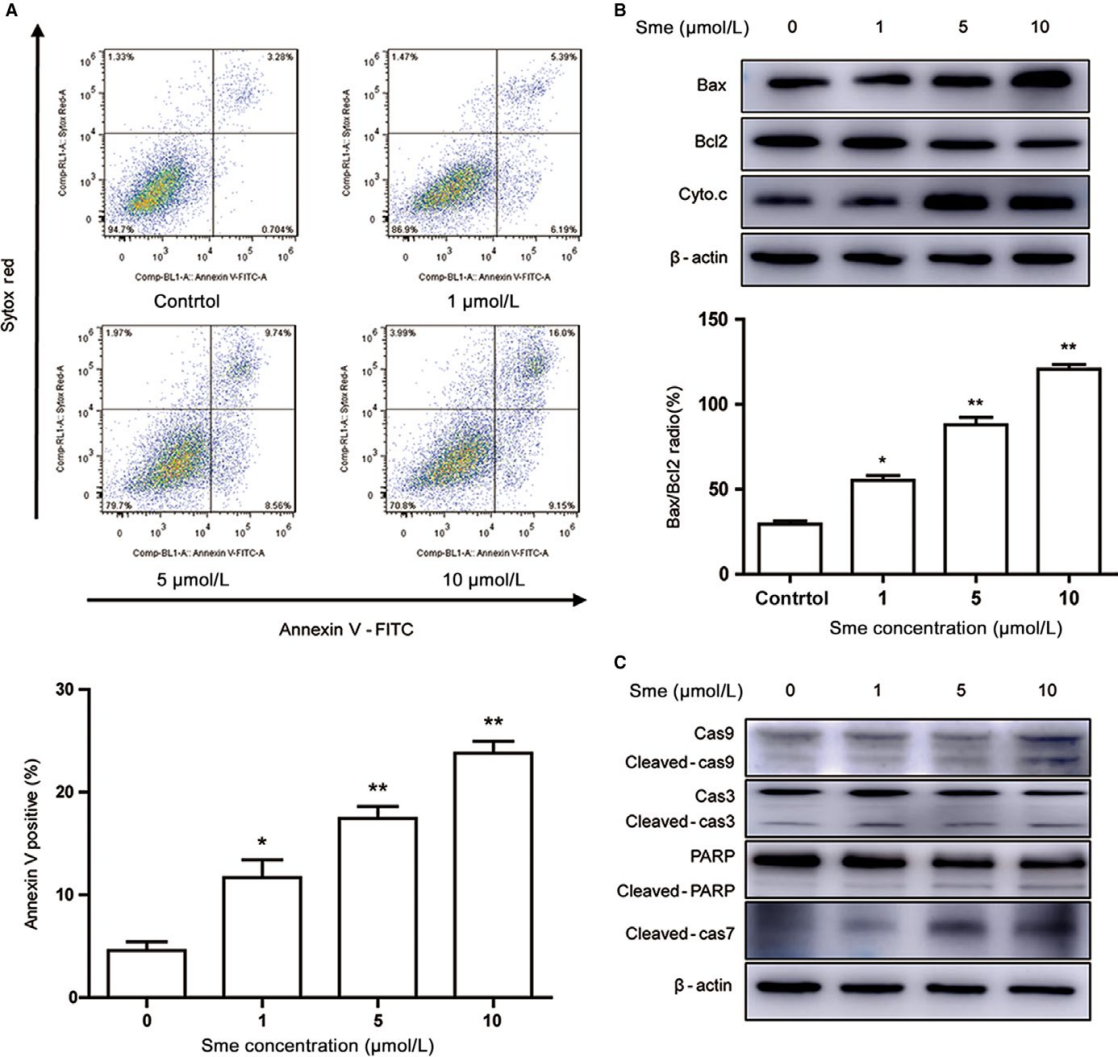
Sme induces apoptosis in MCF7‐Nanog cells. A, Cells were treated with different concentrations of Sme for 36 hours. The apoptotic cells were measured and quantified by flow cytometry using Annexin V‐FITC/Sytox Red staining. B, The expression of Bcl2, Bax, and Cyto.c were detected by western blotting analysis after exposure to Sme for 36 hours, with β‐actin as a loading control. The bar graph represents the ratio of Bax/Bcl2 at the protein levels. Data were expressed as mean ± SD of three experiments. * *P *< .05, ** *P *< .01. C, The expression of caspase‐9, caspase‐3, caspase‐7, and PARP were detected by western blotting analysis after exposure to Sme for 36 hours, with β‐actin as a loading control

DNA damage has been reported to activate the intrinsic apoptosis pathway through the regulation of mitochondria‐related protein. Annexin V‐FITC/Sytox Red double‐staining analysis showed the percentage of the apoptotic cells were dramatically increased after incubated with Sme for 36 hours (Figure 4A). After treatment with Sme, the proapoptotic effector Bax was increased and the antiapoptotic effector Bcl2 was decreased (Figure 4B). Those proteins promoted the release of cytochrome c from the inner mitochondrial membrane to cytosol. We further detected whether mitochondrial dysfunction activated caspase cascade. Figure 4C showed that the expression of cleavage of caspase‐9, ‐3, ‐7 and PARP were dose‐dependent increased after exposure to Sme. The above data revealed that Sme‐induced apoptosis was mediated by the activation of caspase‐dependent intrinsic apoptosis pathway.

### 3.4 Sme induces apoptosis via activating the p38 MAPK and AMPKα pathways

**Figure 5:**
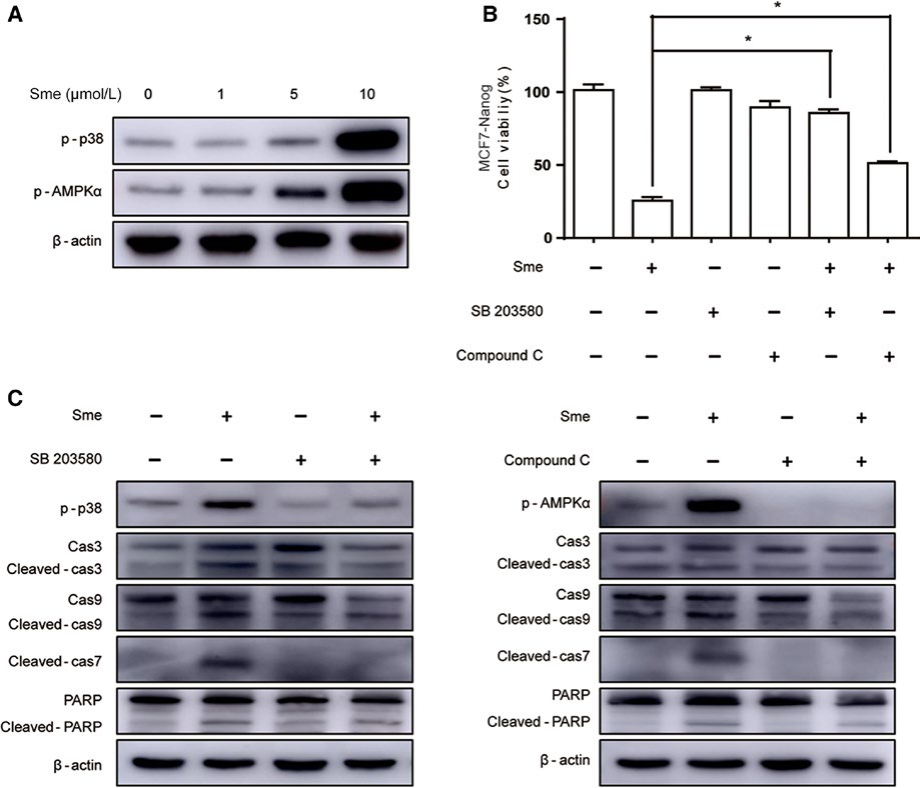
A Sme inhibits cell proliferation via activating the p38 MAPK and AMPKα pathways. A, MCF7‐Nanog cells were treated with Sme at the indicated concentrations for 36 hours, and the expression of p38 and AMPKα was measured by Western blotting. β‐actin served as a loading control. B, MCF7‐Nanog cells were pretreated with 20 μmol/L SB203580 or 1 μmol/L compound C for 4 hours and then exposed to 10 μmol/L Sme for 36 hours. The cell viability was measured by CCK‐8 assay. Data are presented as mean ± SD, * *P *< .05. C, Cells were pretreated with 20 μmol/L SB203580 or 1 μmol/L compound C for 4 hours prior to 10 μmol/L Sme treatment for 36 hours. The expression of caspase‐9, caspase‐3, caspase‐7, and PARP were detected by Western blotting, with β‐actin as a loading control

Considerable researches have shown that the activation of p38 MAPK might inhibit cell growth and induce apoptosis.^19^ AMP‐activated protein kinase (AMPK), the master energy sensor, also plays a crucial role in cell death and apoptosis.^20^ So we speculated whether the activation of p38 MAPK and AMPKα pathways contributed to Sme‐induced apoptosis. As shown in Figure 5A, the phosphorylation level of p38 and AMPKα was markedly increased in MCF7‐Nanog cells after pretreated with Sme for 36 hours. We further applied SB203580 (a p38 MAPK inhibitor) and compound C (an AMPKα inhibitor) to confirm the role of p38 MAPK and AMPKα pathways in the growth inhibitory effect of Sme. MCF7‐Nanog cells were pretreated with 20 μmol/L SB203580 or 1 μmol/L compound C for 4 hours and then inhibitors were discarded. CCK‐8 analysis was conducted after incubation with 10 μmol/L Sme for 36 hours. Figure 5B showed that SB 203580 and compound C significantly abrogated antiproliferative activity of Sme. Western blotting analysis further examined the role of p38 MAPK and AMPKα in Sme‐induced apoptosis. As shown in Figure 5C, SB203580 and compound C obviously decreased the expression of cleaved caspase‐9, ‐3, ‐7 and PARP, which antagonized Sme‐induced apoptosis. Taken together, those data confirmed that Sme induced apoptosis through stimulating the p38 MAPK and AMPKα pathways.

### 3.5 Sme inhibited tumor growth in NOD/SCID mice

**Figure 6:**
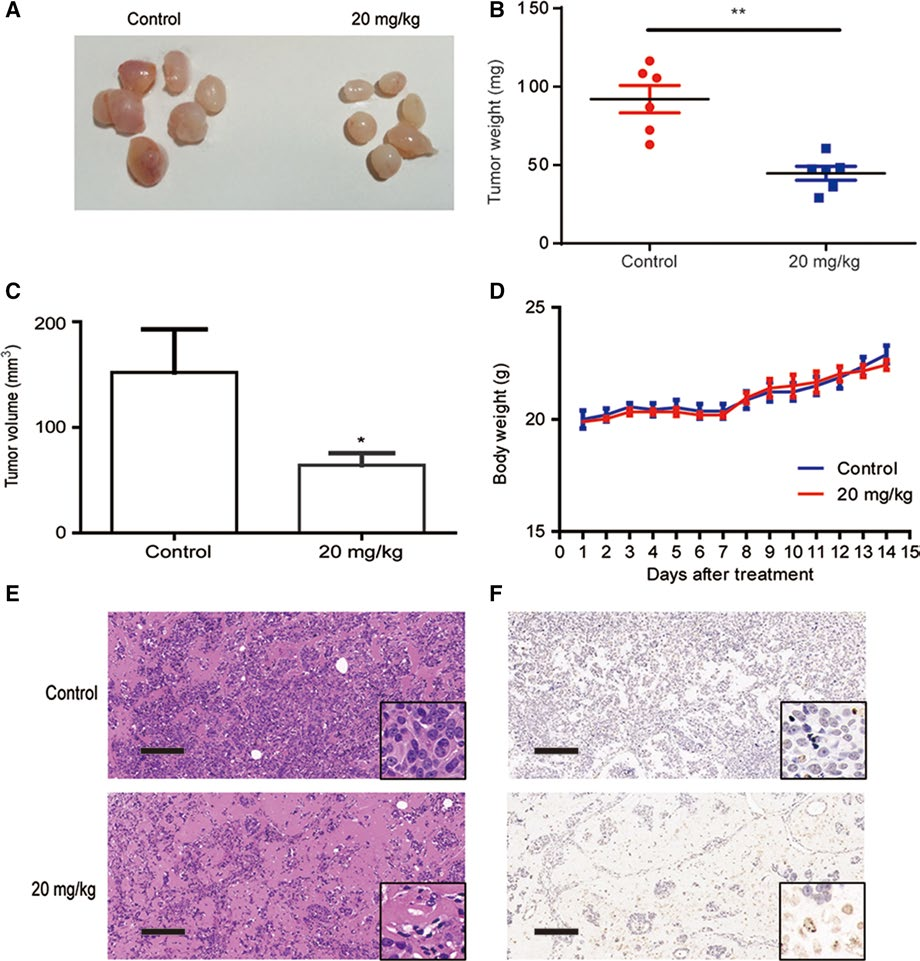
Sme inhibited tumor growth in vivo. MCF7‐Nanog cells were injected into the mammary fat pad of female NOD/SCID mice. Two weeks after injection, the mice were randomly divided into two groups: control and Sme (20 mg/kg), followed by intraperitoneally injection with Sme every other day for two weeks. A, The tumors were surgically excised and photographed two weeks after Sme treatment. B, C, The tumor weight and volume were expressed as mean ± SD. * *P *< .05, ** *P *< .01. D, The body weight was monitored every day after Sme treatment and expressed as mean ± SD. E, The histology was assessed by H&E staining. Scale bar, 100 μm. F, TUNEL assay was used to evaluate apoptotic status of tumor tissues. Scale bar, 100 μm

We established a tumor xenograft model by transplanting MCF7‐Nanog cells into the mammary fat pad of female NOD/SCID mice to confirm the potential antitumor effect of Sme in vivo. Two weeks after injection, the mice were divided into two groups: control and Sme (20 mg/kg), followed by intraperitoneally injection with Sme every other day for two weeks. The results demonstrated that Sme inhibited tumor growth (Figure 6A). Sme significantly decreased the tumor weight (Figure 6B) and tumor volume (Figure 6C) and had no effect on the body weight of the mice (Figure 6D). H&E staining showed that there were many areas of necrosis in tumor tissues of the Sme‐treated group (Figure 6E). However, cancer cells grew luxuriantly with only a few, small focal areas of necrosis in the control group. And more apoptotic cancer cells were positive for TUNEL labeling in tumor tissues of the Sme‐treated group, compared with the control group (Figure 6F). Collectively, those results indicated that Sme inhibited tumor growth in NOD/SCID mice bearing xenografts without overtly toxicity to mice.

## 4 DISCUSSION

Accumulating evidence suggests that CSCs are at the root of inherently tumor‐initiating potential and responsible for cancer relapse and metastasis.^21^ Novel anticancer agents efficiently targeting CSCs are required to eliminate resistance to therapy of cancers. However, the major limitation of identification and characterization of novel agents is the lack of stable and homogeneous CSC‐like cell population in vitro. In this study, we generated a breast CSC‐like model through ectopic overexpression of the stem cell marker Nanog in MCF7 cells and found Sme, a natural compound from marine sponge, preferentially inhibited the growth of these CSC‐like cells in vitro and in vivo.

The recent study indicated that overexpression of Nanog, an essential stem cell markers regulating pluripotency and self‐renewal properties in embryonic stem cells, can promote stem cell characteristics in lung adenocarcinoma (LAC) and hepatocellular carcinoma (HCC).^22^, ^23^ As Nanog has been reported to promote tumorigenesis and metastasis in breast cancer cells, we proposed a hypothesis that overexpression of Nanog might initiate CSC‐like characteristics in breast cancer.^24^ In the present study, we documented that Nanog overexpression initiated typical CSC‐like properties in breast cancer cell line. First of all, overexpression of Nanog promoted the expression of stemness‐associated markers Sox2 and Bmi1, indicating an acquired stem cell characteristic. The dependence on Bmi1 for stem cell maintenance has been confirmed in neurospheres derived from all stages, including embryonic.^25^ Second, MCF7‐Nanog cells exhibited stronger drug resistance to cisplatin, tamoxifen, paclitaxel, and gemcitabine than MCF7‐Ctrl cells, accompanied by enhanced expression of ABC family of multidrug‐resistant genes ABCG2. Third, sphere formation assay showed that ectopically overexpressed Nanog promoted self‐renewal capacity. Finally and most importantly, MCF7‐Nanog cells had the property of more powerful tumorigenicity than MCF7‐Ctrl examined by xenografts in NOD/SCID mice. Those data proposed that our breast cancer cells acquired elevated cancer stem cell properties after ectopic expression of Nanog. This CSC‐like model could be used in vitro to identify agents that selectively kill CSCs.

In our study, three different assays, cell viability, tumor sphere formation, and differentiation, were utilized to analyzed stemness and tumorigenic characteristics of CSC‐like cells. Sme, a sesquiterpene aminoquinone from the marine sponge *Spongia pertusa* Esper, was selectively toxicity against CSC‐like cells. Sox2^26^, ^27^ and Bmi1^28^, ^29^ are crucial pluripotency genes regulating self‐renewal and differentiation in cancer stem cells. Extending these findings, we validated Sme abrogated the self‐renewal ability and prominently suppressed the expression of stemness‐related markers Nanog, Sox2, and Bmi1 in MCF7‐Nanog cells, highlighting its potential anti‐CSC activities. However, Sme did not affect the mRNA level of Nanog (Fig. S4). It has been reported that tumor suppressor p53 represses Nanog expression at a transcriptional level in mouse embryonic stem cells.^30^, ^31^ We documented that Sme might promote the expression of p53, which may result in decreasing expression of Nanog (Fig. S5). Therefore, our current approach can provide an effective option for future development of anti‐CSCs agents.

Several previous studies reported that Sme has multifaceted pharmacological effects on leukemia cells and solid tumors cells. In leukemia cells, Sme extensively involves in promoting erythroid differentiation in human chronic myelogenous leukemia and inhibiting proliferative activity against different leukemia cells through induction of G1 arrest or apoptosis.^32^, ^33^, ^34^ Recent studies have further shown that Sme exhibits antiproliferative and antiangiogenic activities on solid tumor cells.^35^ However, the anticancer mechanism of Sme against CSCs has not been systematically elucidated and still blurred. We here for the first time revealed a unique function of Sme to disrupt self‐renewal pathways and induce intrinsic apoptosis of CSC‐like cells. Induction of cell cycle arrest and apoptosis have become efficient strategies for cancer treatment.^36^ The cell cycle arrest serves as a survival mechanism that allows time and opportunity for the repair of DNA. Once DNA repair is failed, the apoptotic cascade will be activated and finally cause cell death.^37^ In breast CSC‐like cells, we found that Sme caused DNA damage, cell cycle arrest, and apoptosis. Sme led to a decrease in the level of cell cycle regulatory molecules of G0/G1 checkpoint. Simultaneously, Sme induced apoptosis via strongly activating the p38 MAPK and AMPKα pathways in MCF7‐Nanog cells.

There is growing evidence supporting that p38 MAPK and AMPK play crucial roles in cancer cell growth, proliferation, survival.^38^, ^39^, ^40^ Meng et al^41^ reported that p38 MAPK exhibits overexpression in highly metastatic human and mouse breast cancer cell lines and promotes basal‐like and metastatic properties in breast tumor samples. Mei et al^42^ further showed that p38 MAPK activation contributes to an increase in CSC population and metastasis. It has been reported that radioresistant glioblastoma stem cells acquired enhanced AMPK activity and in normal human mammary epithelial cells AMPK mediated the mammosphere formation, indicating that AMPK might involve in regulating the self‐renewal and therapeutic resistance of CSCs.^38^, ^39^ Our study showed that Sme induced apoptosis via activating the p38 MAPK and AMPKα pathways in MCF7‐Nanog cells. We further compared the phosphorylated level of p38 and AMPKα between MCF7‐Ctrl cells and MCF7‐Nanog cells after incubation with Sme. Western blotting analysis displayed that the expression of the phosphorylation level of p38 and AMPKα in MCF7‐Nanog cells was significantly higher than that in MCF7‐Ctrl cells (Fig. S6), which could explain why MCF7‐Nanog cells were more sensitive to Sme and account for the preferential antitumor activity of Sme against cancer stem‐like cells. P38 MAPK inhibitor SB203580 and AMPKα inhibitor compound C repressed Sme‐induced apoptosis and reversed activation of caspase pathway. These results support that p38 MAPK and AMPKα might function as promising therapeutic targets in breast CSCs. An improved understanding of the molecular link between Sme and its regulation of CSC properties revealed by our current study will further clarify the molecular signature of cancer stem cells.

Herein, we established a breast CSC‐like model though overexpression of Nanog in MCF7 cells. This approach creates a stable and homogeneous CSC‐like cell population and can be applied to drug screening for more selective anti‐CSC agents. Additionally, we validated that Sme exhibits preferential antitumor effects on MCF7‐Nanog cells in vitro and in vivo. These investigations provide insight into antitumor mechanisms of Sme and provide a promising natural agent to breast cancer treatment.

## DISCLOSURE STATEMENT

Authors declare no conflict of interests for this article.
