# Supplementary figures and images for: Marine sponge‐derived smenospongine preferentially eliminates breast cancer stem‐like cells via p38/AMPKα pathways
Source: Cancer Med. 2018 Jul 7;7(8):3965–76. doi: 10.1002/cam4.1640 (PMC6089165; doi:10.1002/cam4.1640)

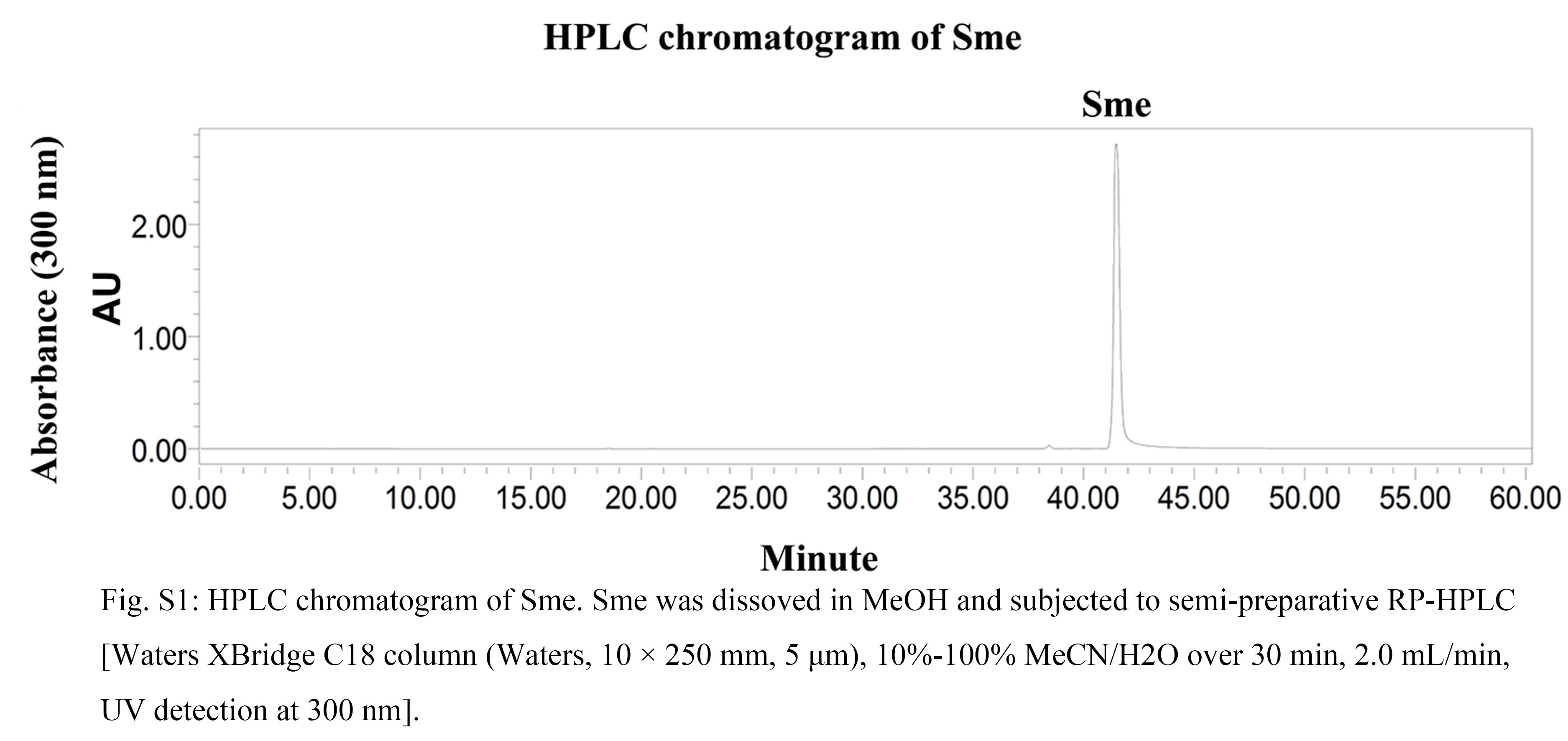

Supplement: Supplementary file 1 [file CAM4-7-3965-s001.jpg]

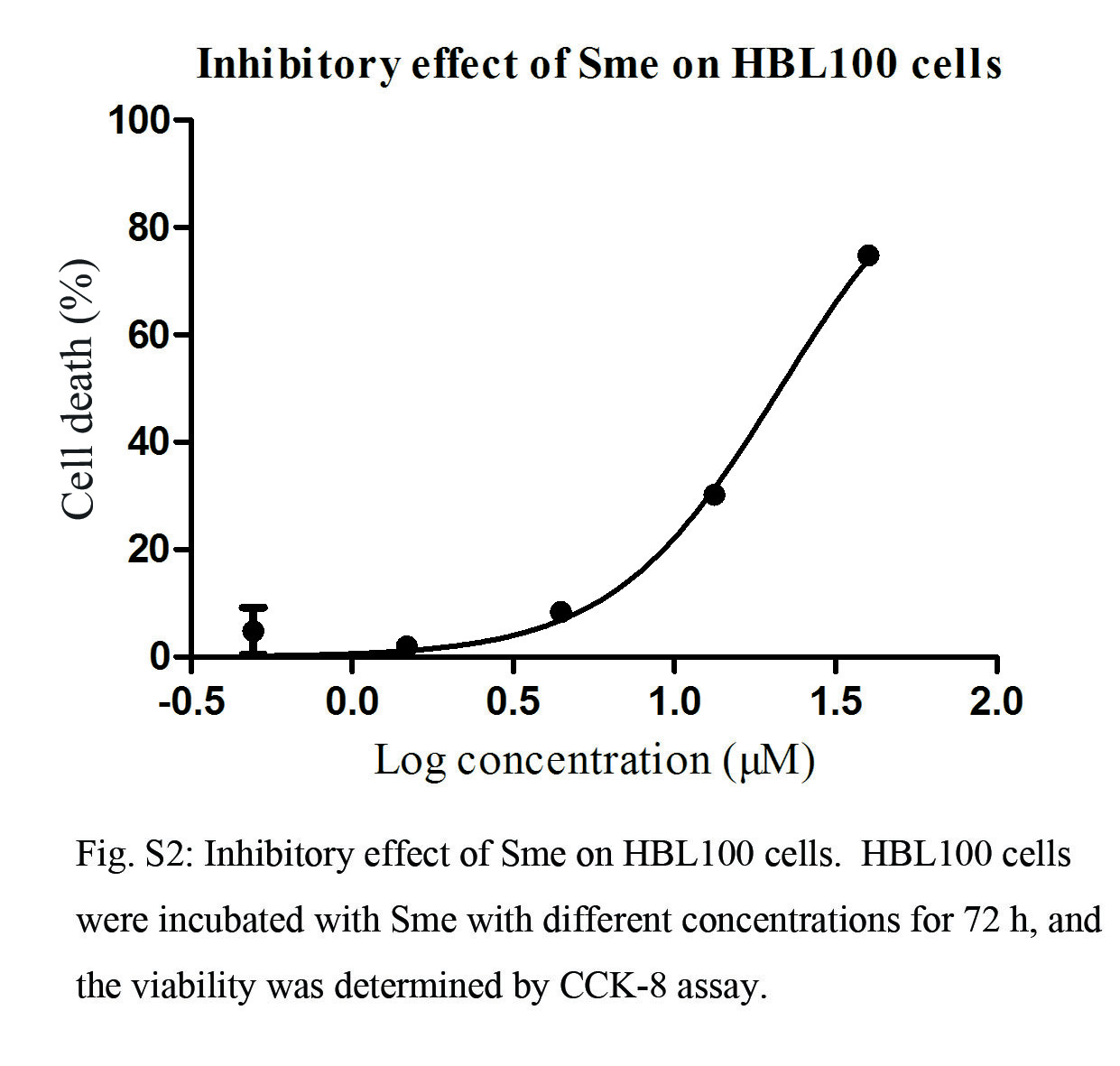

Supplement: Supplementary file 2 [file CAM4-7-3965-s002.jpg]

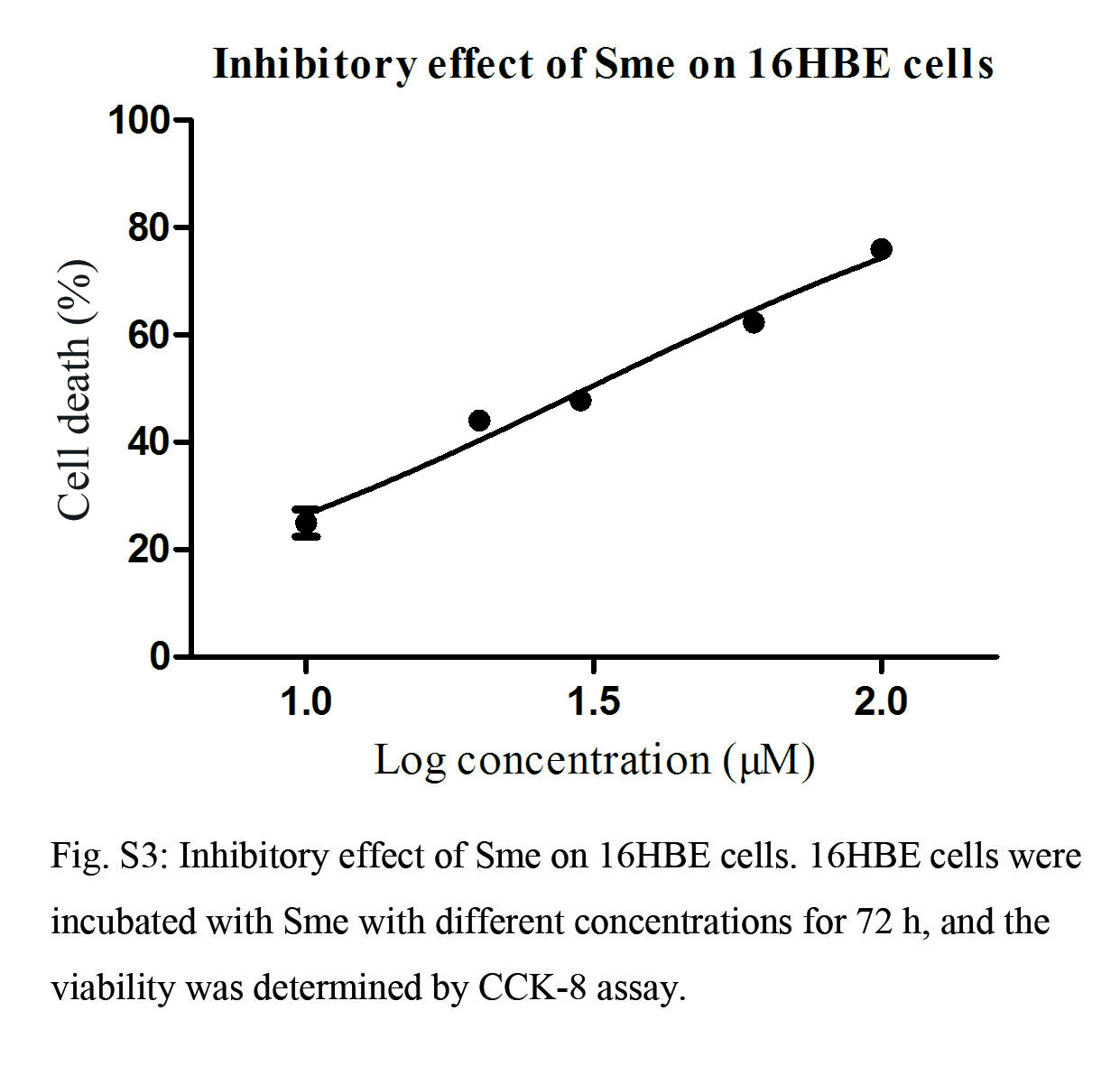

Supplement: Supplementary file 3 [file CAM4-7-3965-s003.jpg]

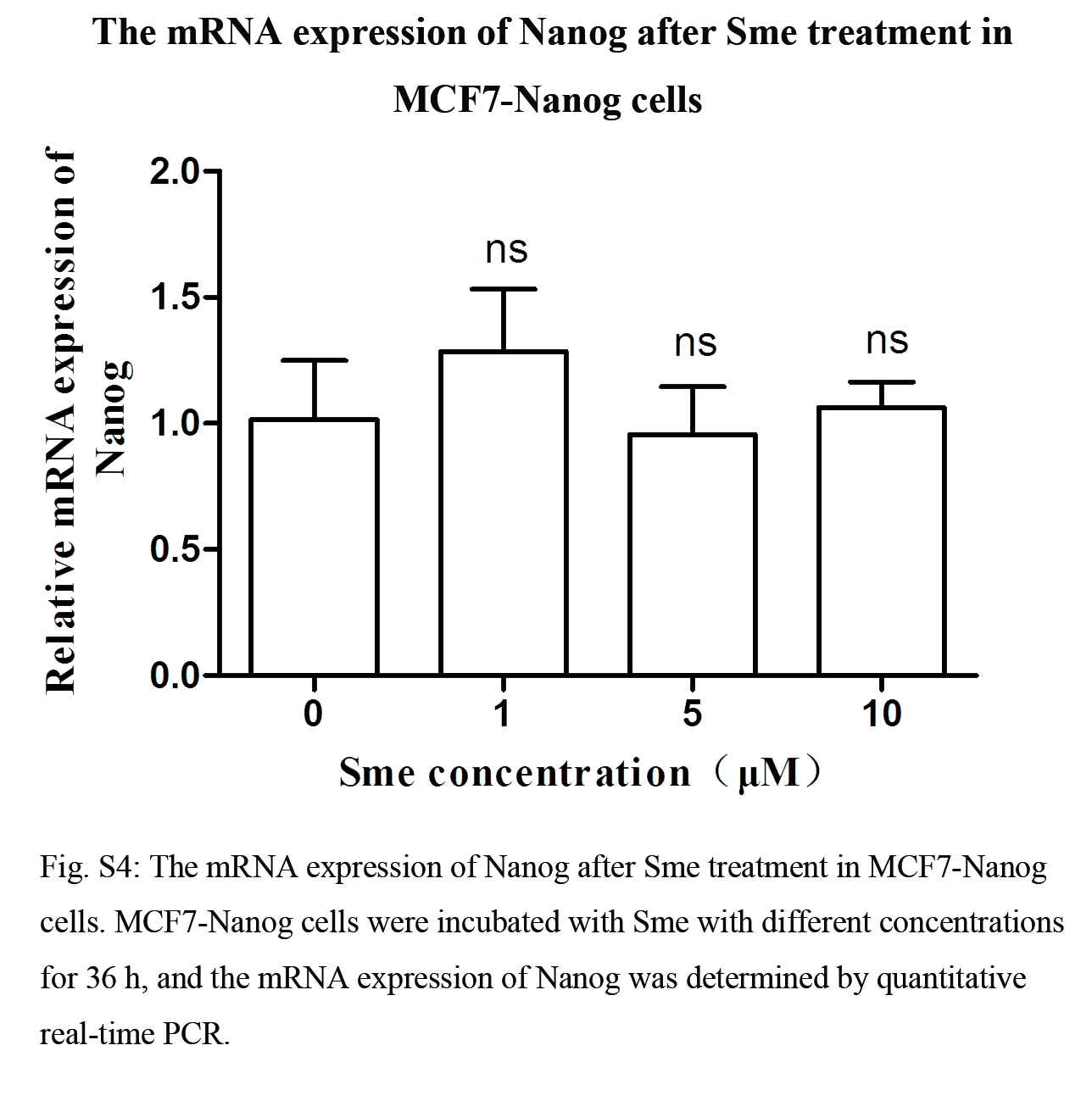

Supplement: Supplementary file 4 [file CAM4-7-3965-s004.tif]

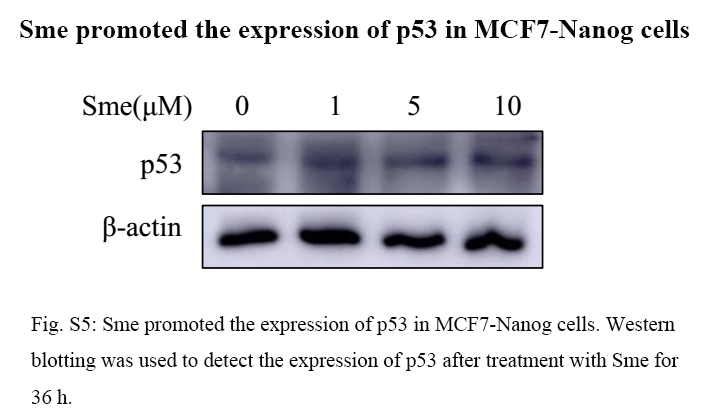

Supplement: Supplementary file 5 [file CAM4-7-3965-s005.tif]

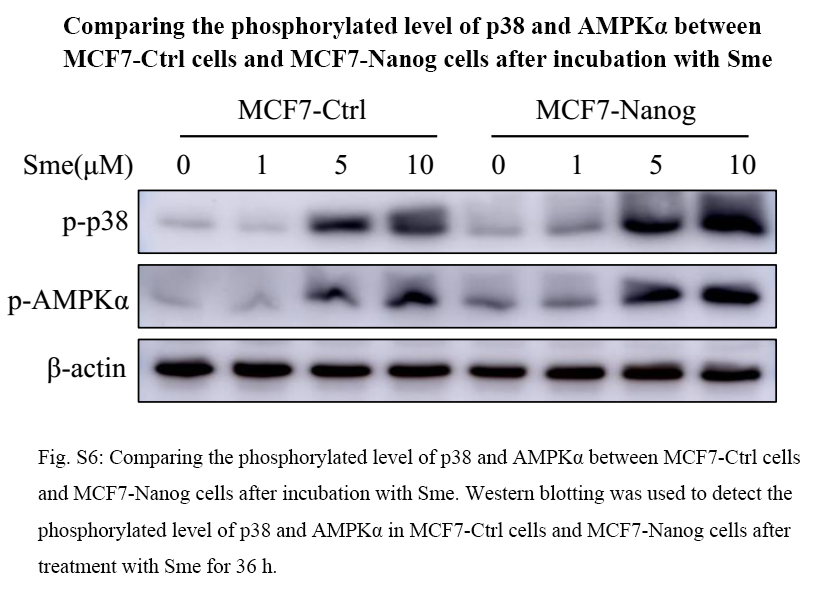

Supplement: Supplementary file 6 [file CAM4-7-3965-s006.tif]

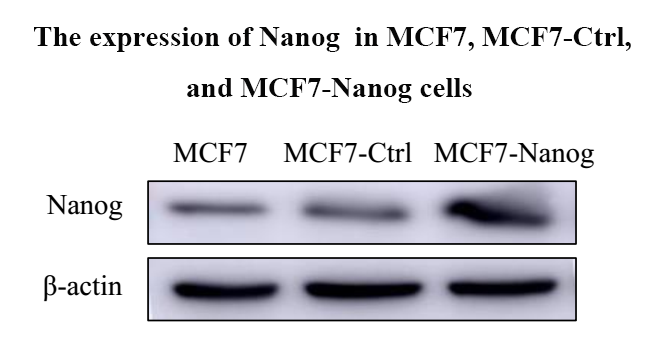

Supplement: Supplementary file 7 [file CAM4-7-3965-s007.tif]
