# Supplementary material for: Marine sponge‐derived smenospongine preferentially eliminates breast cancer stem‐like cells via p38/AMPKα pathways
Source: Cancer Med. 2018 Jul 7;7(8):3965–76. doi: 10.1002/cam4.1640 (PMC6089165; doi:10.1002/cam4.1640)
Supplement: Supplementary file 8 [file CAM4-7-3965-s008.docx]

Table S1. List of primers used in RT-qPCR ( from 5′ to 3′)

| Oct4 | forward | CTGGGTTGATCCTCGGACCT |
| --- | --- | --- |
|  | reverse | CCATCGGAGTTGCTCTCCA |
| CD133 | forward | AGTCGGAAACTGGCAGATAGC |
|  | reverse | GGTAGTGTTGTACTGGGCCAAT |
| CD44 | forward | CTGCCGCTTTGCAGGTGTA |
|  | reverse | CATTGTGGGCAAGGTGCTATT |
| ALDH1 | forward | TCCAGCCCACAGTGTTCTCTAAT |
|  | reverse | GATTTGCTGCACTGGTCCAA |
| Nestin | forward | ACCCTTCCAGACTCCACTC |
|  | reverse | CACTCCTCTTCTCCCTCCTC |
| Sox2 | forward | GCCGAGTGGAAACTTTTGTCG |
|  | reverse | GGCAGCGTGTACTTATCCTTCT |
| KIF4 | forward | TACTGCGGTGGAGCAAGAAG |
|  | reverse | CATCTGCGCTTGACGGAGAG |
| Musashi-1 | forward | TCCCTCGGCGAGCACA |
|  | reverse | GACAGCCCCCCCACAAA |
| EPCAM | forward | CAGTTGGTGCACAAAATACTGTCA |
|  | reverse | CCATTCATTTCTGCCTTCATCA |
| Bmi1 | forward | CAACTGGTTCGACCTTTGCAGATA |
|  | reverse | GATGTGCCAATTGCTTCTAATGGA |
| ABCG2 | forward | GGATGAGCCTACAACTGGCTT |
|  | reverse | TTCCTGAGGCCAATAAGGTG |
| Cyclin E1 | forward | AAGGAGCGGGACACCATGA |
|  | reverse | ACGGTCACGTTTGCCTTCC |
| CDK4 | forward | CAGCTTGCCCGAGTTCTACT |
|  | reverse | TGTCCTCAGAGTTAGCCGGA |
| β-actin | forward | CCTGGCACCCAGCACAAT |
|  | reverse | GGGCCGGACTCGTCATAC |
