# Supplementary material for: Marine sponge‐derived smenospongine preferentially eliminates breast cancer stem‐like cells via p38/AMPKα pathways
Source: Cancer Med. 2018 Jul 7;7(8):3965–76. doi: 10.1002/cam4.1640 (PMC6089165; doi:10.1002/cam4.1640)
Supplement: Supplementary file 9 [file CAM4-7-3965-s009.docx]

Table S2. In vivo tumorigenicity experiments of MCF7, MCF7-Ctrl and MCF7-Nanog cells.

In vivo tumorigenicity experiments

| **Days** | **0** | **6** | **9** | **12** |
| --- | --- | --- | --- | --- |
| MCF7 | 0/5 | 0/5 | 3/5 | 5/5 |
| MCF7-Ctrl | 0/5 | 0/5 | 3/5 | 5/5 |
| MCF7-Nanog | 0/5 | 2/5 | 5/5 | 5/5 |
